# Latest Advances in Pancreatobiliary Endoscopy

**DOI:** 10.3390/medicina61030550

**Published:** 2025-03-20

**Authors:** Marco Spadaccini, Alessandro Fugazza

**Affiliations:** 1 Department of Biomedical Sciences, Humanitas University, 20072 Pieve Emanuele, Italy; 2 Endoscopy Unit, Humanitas Clinical and Research Center—IRCCS, 20089 Rozzano, Italy; alessandro.fugazza@humanitas.it

The field of biliopancreatic endoscopy has witnessed transformative advancements, driven by technological innovations, novel techniques, and an evolving understanding of disease processes. This Special Issue showcases cutting-edge research and developments that push the boundaries of clinical practice and address longstanding challenges. Endoscopic ultrasound (EUS)-guided fine-needle biopsy (FNB) has emerged as the preferred technique for sampling solid pancreatic lesions [1,2]. By delivering larger tissue cores and preserving histological architecture, FNB enhances diagnostic accuracy and facilitates personalized treatments. This approach surpasses fine-needle aspiration (FNA) and demonstrates the critical role of needle design, sampling techniques, and specimen processing in optimizing outcomes. The review by Dhar et al. [3] offers insights to help endoscopists navigate these advanced methodologies effectively.

Interventional EUS has expanded beyond diagnostics to manage complications of pancreatic cancer, including gastric outlet obstruction and malignant biliary obstruction [4,5,6]. Unresectable malignant gastric outlet obstruction, a significant therapeutic challenge, is now addressed using minimally invasive techniques such as laparoscopic procedures, endoscopic stenting (ES), and EUS-guided gastroenterostomy (EUS-GE). EUS-GE combines the benefits of ES’s minimally invasive nature with the durability of surgical solutions, providing a promising alternative for palliation as presented by Fugazza et al. [7]. Similarly, EUS-guided choledochoduodenostomy (EUS-CDS) with electrocautery-enhanced lumen-apposing metal stents (EC-LAMS) is redefining the management of distal malignant biliary obstructions. Traditionally reserved for ERCP failures, EUS-CDS is now being positioned as a first-line intervention in both palliative and preoperative settings, reducing the risk of iatrogenic pancreatitis and postoperative complications. This innovation challenges ERCP’s dominance as underlined in the review by Guilmoteau et al. [8], urging further comparative studies to cement its clinical role. Biliary drainage in patients with surgically altered anatomy (SAA) poses unique challenges. An Italian survey highlighted in this Issue reveals variability in clinical practices, especially for Roux-en-Y reconstructions [9]. While EUS-guided interventions are gaining traction as rescue modalities [10,11], percutaneous drainage remains a common first-line option. Our findings emphasize the need for standardized protocols and greater collaboration between tertiary and non-tertiary centers to improve outcomes.

Apart from biliary drainage, EUS-guided drainage has become a cornerstone in managing pancreatic fluid collections (PFCs), particularly infected pancreatic necrosis [12,13]. However, unresolved questions remain about timing, stent selection, and procedural techniques. Lumen-apposing metal stents (LAMSs) offer direct access for necrosectomy but are associated with significant complications. This issue delves into these debates, providing a comprehensive analysis of current practices and identifying areas for further research [14]. In particular, complications like arterial bleeding, a potentially life-threatening issue during endoscopic necrosectomy, are also explored. A featured case study [15] demonstrates the effectiveness of the Coagrasper in achieving hemostasis, while cautioning against overuse to avoid exacerbating vessel damage. This case underscores the importance of precision and judicious tool application in complex scenarios.

Other benign conditions have also benefited from the EUS revolution. Acute cholecystitis in patients unfit for surgery necessitates innovative drainage solutions. The review by Troncone et al. [16] highlights transpapillary and EUS-guided approaches, showcasing LAMS technology’s role in enabling not only decompression but also cholecystoscopy-guided interventions such as gallstone lithotripsy. Despite these advancements, uncertainties remain, including the comparative efficacy of different techniques and the long-term outcomes of EUS-guided methods.

Finally, the Issue also addresses longstanding challenges like managing complex biliary stones [17,18,19,20,21]. Choledocholithiasis, a common indication for ERCP, often involves difficult stones requiring advanced techniques such as large-balloon papillary dilation, mechanical lithotripsy, and laser lithotripsy. The narrative review by Manti et al. [22] synthesizes current evidence to offer a roadmap for effective management, emphasizing tailored approaches to optimize therapeutic outcomes.

This Special Issue highlights the vibrant innovation and collaboration that propel the field of biliopancreatic endoscopy forward. From refining diagnostic tools to pioneering therapeutic interventions, the included articles provide a comprehensive snapshot of the latest advancements. As the field evolves, continued emphasis on rigorous research, multidisciplinary collaboration, and patient-centered care remains essential. We hope this collection inspires and informs practitioners and researchers committed to advancing this critical area of medicine.
